# Effect of Processing, Post-Harvest Irradiation, and Production System on the Cytotoxicity and Mutagenicity of *Vitis labrusca* L. Juices in HTC Cells

**DOI:** 10.1371/journal.pone.0107974

**Published:** 2014-09-22

**Authors:** Elisângela Düsman, Igor Vivian de Almeida, Luciano Lucchetta, Veronica Elisa Pimenta Vicentini

**Affiliations:** 1 Department of Biotechnology, Genetics and Cell Biology, State University of Maringá, Paraná, Brazil; 2 Federal Technological University of Paraná, Francisco Beltrão, Paraná, Brazil; University of Science and Technology of China, China

## Abstract

The juices of grapes (*Vitis labrusca* L.) are similar to the fruit itself because the main constituents of the fruit are present in the juice. However, their quality characteristics may be modified by the harsh technological processes used for the production of integral food, such as production systems of raw materials and post-harvest treatment of grapes with ultraviolet (UV) irradiation. Therefore, the present study analyzed juices produced naturally (by liquefying the fruit) or by the technological process of extraction by steam distillation (90°C) of grapes from organic and conventional production systems that were untreated or treated with UV type C (65.6 J/m^2^ for 10 minutes). Using cultures of *Rattus norvegicus* hepatoma cells (HTC) in vitro, cytotoxic effects were assayed by the MTT test and by calculating the cytokinesis blocked proliferation index (CBPI), and mutagenic effects were measured by the cytokinesis block micronucleus assay. The results of the MTT assay and the CBPIs indicated that none of the juices were cytotoxic, including those that induced cell proliferation. The results of the micronucleus assay showed that none of the juices were mutagenic. However, the average number of micronuclei was lower in the juices produced from organic grapes, and cell proliferation, soluble acids and phenolic compounds were significantly higher. Compared with the natural juices, the integral juices of conventional grapes showed a higher average number of micronuclei as well as lower stimulation of cell proliferation and lower levels of bioactive compounds. The results demonstrate a beneficial effect of UV-C irradiation of post-harvest grapes in stimulating the synthesis of nutraceutical compounds without generating cytotoxic or mutagenic substances. Taken together, our findings support the consumption of grape juice and the application of food production techniques that enhance its nutritional value and promote its production, marketing and consumption.

## Introduction

Functional food is any food or ingredient that, beyond its basic nutritional functions, produces metabolic and/or physiological effects that are beneficial to health when consumed in the usual diet [1]. Grapes (*Vitis spp*.) are included in this class and possess many medicinal properties. They can be used to treat infections [2], cardiovascular diseases [3] and hyperglycemia [4], and they can serve as chemopreventive [5], chemotherapeutic [6], antioxidant [7] and antimutagenic [8] agents.

Grape juices are nutritionally similar to the grape itself because they contain the fruit's main constituents. In addition to water, grape juice consists of high levels of sugar; nitrogenous compounds, such as amino acids, polypeptides and proteins; phenolic compounds, such as anthocyanins, tannins and phenolic acids; tartaric acid; malic acid; citric acid; minerals, such as potassium, calcium, magnesium, phosphorus, manganese, iron, copper, zinc, lithium and rubidium; and vitamins, including thiamine, riboflavin, niacin, ascorbic acid and inositol [9]–[11].

However, some harsh technological processes can cause extensive changes in food that modify its quality characteristics. Some changes are desirable, such as inactivation of antinutritional factors by heat and creation of flavors. However, other changes are undesirable and include loss of vitamins or color, changes in texture, and production of substances that interfere with aroma [12], [13].

In general, organic products are perceived by the public as safer and healthier than those produced by conventional agriculture. Stemmed plants in organic farming usually have a longer ripening period than conventional plants, and as flavonoids are formed during this period, it is believed that organic production systems yield plants with a higher phenolic content [14].

Furthermore, the synthesis of phenolic compounds, particularly resveratrol, can be increased by exposure to ultraviolet (UV) irradiation. The synthesis of resveratrol in grapes occurs in response to stress caused by fungal attack (e.g., *Botrytis cinerea* and *Plasmopara viticula*), mechanical damage or UV irradiation [15].

According to Versari et al. [16], type C UV irradiation is an important factor that can act as a switch, controlling the expression of specific genes involved in cell growth and secondary metabolism in plants. However, it is noteworthy that exposure of plants to high levels of UV irradiation can cause cell damage and oxidative stress [17], leading to DNA fragmentation and programmed cell death [18].

Food intake is a major route of human exposure to different compounds, some of which can induce DNA mutations and promote tumor development. Therefore, to promote safe food consumption, it is important to assess the risk associated with the consumption of juice from grapes (*Vitis labrusca* L.) that are produced by natural and technological processes, with and without exposure to UV-C, from organic and conventional production systems.

## Materials and Methods

### Cell Line – HTC

HTC cells derived from a *Rattus norvegicus* hepatoma were obtained from the Rio de Janeiro Cell Bank in Rio de Janeiro, Brazil. The cells were grown in 25 cm^2^ culture flasks containing 10 mL of DMEM culture medium (Invitrogen, Carlsbad, CA, USA) with 0.01 mL of streptomycin and penicillin, supplemented with 10% fetal bovine serum (Invitrogen, Carlsbad, CA, USA) and incubated at 37°C in BOD incubator. The cell cycle in this cell line is approximately 24 hours.

### Preparations of *Vitis labrusca* L. juices

The juices used for treatment were prepared from grapes of the *Vitis labrusca* L. Concord variety from the 2012 crop that was cultivated in the municipality of Verê in the microregion of Francisco Beltrão in southwest Paraná, Brazil. The grapes were obtained from two nearby properties (5.3 km apart): one was conventionally cultivated (564 m altitude, latitude 25° 54′ 01″ S and longitude 52° 53′ 51″ W), and the other was used for organic farming (492 m altitude, latitude 25° 51′ 21″ S and longitude 52° 55′ 06″ W). The properties were exposed to similar climatic and edaphologic conditions.

The conventional system employed chemical soil fertilization and fungicides for controlling fungi. In the organic system, the vineyard had a green roof soil, and copper sulfate, calcium hydroxide and sulfur were used for disease control.

The grapes were selected according to their degree of sanity, washed in chlorinated water (hypochlorite, 50 mg/L sodium, pH 5.0) and stored under refrigeration (±5°C). Half of the grapes collected from each culture system were submitted to UV-C treatment, as described by Cantos et al. [19], with a radiation fluency rate of 65.6 J/m^2^ at a distance of 30 cm from the light source. The grapes were arranged in a single layer on trays that were irradiated for 5 minutes in a cabin equipped with three UV-C lamps (90 W). The bunches were inverted by 180°, and the light source was maintained for 5 additional minutes; the total irradiation time was 10 minutes. The irradiated material was stored for 3 days at 25±5°C with 70% humidity in the absence of light to promote the biosynthesis of bioactive compounds.

The grapes from organic and conventional farming that were treated and untreated with UV-C were used for obtaining integral and natural juices. The integral juice was obtained through extraction by steam distillation at 90°C, packaged in a glass container and stored in the dark. The natural juice was prepared by liquefying the grapes, as performed by general consumers.

The physico-chemical analysis of grapes and juices was performed by Pinto [20], emphasizing the evaluation of the level of soluble acids, total phenolic compounds, anthocyanins, trans-resveratrol and antioxidant activity.

### Ethics Statements

The grapes used in this study were provided by farmers, who gave permission to conduct the study on their private land. The field studies did not involve endangered or protected species.

### Cytotoxicity Assay (MTT)

The MTT [3-(4,5-Dimethylthiazol-2-il)-2,5-diphenyl tetrazolium bromide] cytotoxicity assay was performed following the protocol suggested by Mosmann [21]. Cell culture plates (96 wells) were used, and in each well 2.0×10^4^ cells were seeded; the control wells contained no cells (blank). The cells were cultured for 24 hours with 150 µL of culture medium. Afterwards, the culture medium was removed from the plate, and 150 µL of one of the following treatment media was added: negative control (20 µL of phosphate buffered saline (PBS)/mL culture medium), the cytotoxic agent methyl methanesulfonate (MMS) (final concentration of 150 µM) and eight different grape juices at concentrations of 2, 10, 20, 30 and 40 µL/mL culture medium (natural or integral juice from organic grapes, conventional grapes, organic grapes treated with UV-C and conventional grapes treated with UV-C). To the blank wells, the highest concentration of each juice was added.

The cells were incubated for 24 hours, and the culture medium was then replaced by 150 µL of serum-free medium containing MTT at a concentration of 0.167 mg/mL. The plate was incubated for 4 additional hours, followed by removal of the MTT-containing medium and addition of 150 µL of dimethylsulfoxide (DMSO) to the wells to dilute the formazan crystals formed. The reading was performed in a microplate reader (FlexStation) at 550 nm, and the data are expressed as average absorbance values from three biological replicates.

### Cytokinesis Block Micronucleus Assay

Cells (10^6^) were cultured for 24 hours in flasks containing 5 mL of culture medium. After one cell cycle, the cells were incubated with Cytochalasin-B (98%, CAS 14930-96-2, Sigma, St. Louis, MO, USA) at a final concentration of 3.0 µg/mL DMEM for 26 hours and with the treatment solutions (for 24 hours) to assess cytotoxicity and mutagenicity, as described by Fenech [22]. The treatment groups were the following: negative control (20 µL of phosphate buffered saline (PBS)/mL culture medium), the mutagen doxorubicin (98%, CAS 25316-40-9, Ackros, New Brunswick, USA) at a final concentration of 0.2 µg/mL culture medium, and eight different grape juices at concentrations of 2, 10 and 20 µL/mL culture medium (natural or integral juice from organic grapes, conventional grapes, organic grapes treated with UV-C and conventional farming grapes treated with UV-C).

The cells were collected according to the protocol of Salvadori et al. [23]. They were first trypsinized at 37°C (500 µL of trypsin with 0.025% EDTA (Gibco, Carlshad, CA, USA)) and then prefixed with a drop of formaldehyde, centrifuged (5 minutes at 1,000 rpm), hypotonized (1.5 mL sodium citrate 1%), centrifuged again and fixed (5 mL of a 3∶1 mixture of methanol:acetic acid). The slides were prepared by dispensing a droplet of fixative onto the collected material, coating with a film of cold distilled water, and staining with Giemsa (5%). The slides were then stored in the refrigerator.

**Figure 1:**
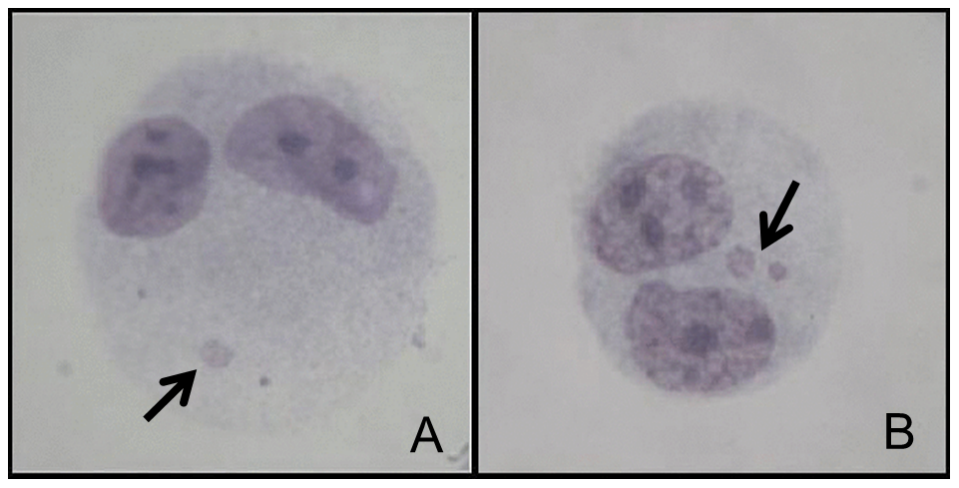
HTC binucleated cells with micronuclei (arrow) (A and B).

**Figure 2:**
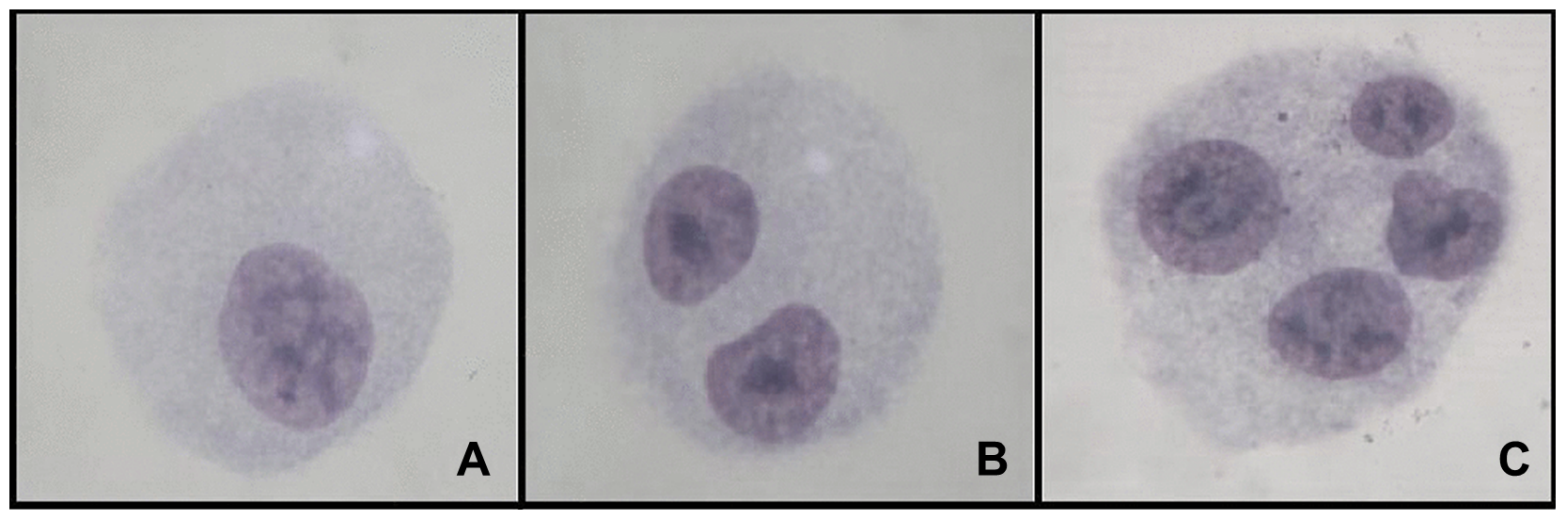
Mononucleated (A), binucleate (B) and multinucleate HTC cells (C).

The experiments were performed in three biological replicates, and the slides were analyzed according to the criteria established by Fenech [22]. To assess the frequency of cells with micronuclei (MN), 1,000 binucleated cells were counted per replicate, totaling 3,000 binucleated cells, as showed in Figure 1. To determine the Cytokinesis Blocked Proliferation Index (CBPI), 500 cells were counted per replicate, totaling 1,500 cells per group, to score the number of mono-, bi- and multinucleated cells, as showed in Figure 2. The CBPI was calculated using the following formula: CBPI  =  [(N° mononucleated cells) + (N° binucleate cells ×2) + (N° multinucleate cells ×3)]/total number of cells. The percentage of cytostasis [24] was calculated as follows: % Cytostasis  = 100–100 [(CBPI_Treatment_ – 1) ÷ (CBPI_Control_ – 1)].

### Statistical analysis

Statistical analysis of the average absorbance values obtained by the MTT assay, the average number of binucleated cells with micronuclei and the CBPI was performed by analysis of variance (one-way ANOVA) followed by the Tukey test (α = 0.05, p<0.05, n = 3) using the GraphPad InStat Program.

## Results and Discussion

The juices of grapes (*Vitis labrusca* L.) produced by conventional and organic farming (Table 1) were not cytotoxic by the MTT assay using HTC cells treated for 24 hours. Similar results were shown by Charles et al. [25], wherein green grapes were not cytotoxic to Chinese hamster ovary cells (CHO-K1-BH4).

**Table 1:** Average value and standard deviation (SD) of the absorbance in the MTT cytotoxicity test using HTC cells treated with methyl methanesulfonate (150 µM) or integral and natural juices of conventional and organic grapes at concentrations 2, 10, 20, 30 and 40 µL/mL and untreated or treated with UV-C or the negative control group (20 µL PBS/mL).

Groups			Percentage of Cell Proliferation ±SD)	
			Conventional Grapes	Organic Grapes
Control			100±9.89	100±26.31
Methyl methanesulfonate			16.48±2.19^a^	26.31±3.50^a^
		2 µL/mL	69.23±12.08	82.45±14.03
		10 µL/mL	103.29±26.37	110.52±14.03
	Untreated UV-C	20 µL/mL	101.09±27.47	133.33±21.05
		30 µL/mL	123.37±30.76	156.14±43.85^b^
Integral Juice		40 µL/mL	126.37±40.65^b^	149.12±49.12^b^
		2 µL/mL	118.68±24.17	142.10±29.82
		10 µL/mL	116.48±20.87	143.85±24.56
	Treated UV-C	20 µL/mL	124.17±31.86	121.05±22.80
		30 µL/mL	115.38±28.57	107.01±22.80
		40 µL/mL	129.67±26.37	108.77±42.10
Control			100±16.16	100±18.36
Methyl methanesulfonate			19.19±2.02^a^	16.32±2.04^a^
		2 µL/mL	114.14±29.29^c^	71.42±8.16
		10 µL/mL	128.28±23.23	116.32±15.30^e^
	Untreated UV-C	20 µL/mL	132.32±23.23	128.57±15.30^e^
		30 µL/mL	134.34±23.23	133.67±16.32^e^
Natural Juice		40 µL/mL	134.34±18.18	143.87±13.26^ae^
		2 µL/mL	55.55±22.22	112.24±23.46^e^
		10 µL/mL	90.90±37.37	125.51±18.36
	Treated UV-C	20 µL/mL	108.08±35.35^c^	129.59±15.30
		30 µL/mL	129.29±32.32^c^	129.59±19.38
		40 µL/mL	138.38±18.18^cd^	127.55±22.44

n = 3; 2.0×10^4^ cells per well.

a Statistically significant result relative to the negative control.

b Statistically significant result relative to the treatment with 2 µL/mL integral juice of UV-untreated grapes.

c Statistically significant result relative to the treatment with 2 µL/mL natural juice of UV-C-treated grapes.

d Statistically significant result relative to the treatment with 10 µL/mL natural juice of UV-C-treated grapes.

e Statistically significant result relative to the treatment with 2 µL/mL natural juice of UV-untreated grapes.

On the other hand, treatment with natural juice from organic UV-untreated grapes at a concentration of 40 µL/mL yielded a significantly higher average absorbance compared with the negative control (40 µL/mL = 1.41±0.13, CO = 0.98±0.18) (Table 1). In addition, all groups showed higher average absorbance values than the treatments with the cytotoxic agent MMS. These results indicate that the tested grape juices have stimulated the proliferation of HTC cells because, according to Mosmann [21], the amount of formazan produced by mitochondrial enzymes from the tetrazolium salt (MTT) and detected by the absorbance assay is directly proportional to the number of living cells (that is, cells with active mitochondria). Thus, because only living cells have mitochondrial activity, the level of mitochondrial activity indicates cell viability [26].

The stimulation of cell proliferation may be due to the high content of vitamins [9]–[11] and sugar in natural juices and in integral juices (data published by Pinto [20]). According to Guéant et al. [27] and Wang et al. [28], vitamins at adequate concentrations act as promoters of cell growth and proliferation and enhance DNA synthesis.

The lowest juice concentration tested (2 µL/mL), and thus the smallest amount of vitamins and sugar to which the cells were exposed, resulted in lower absorbance values in the MTT assay. Therefore, a dose-response effect was observed, wherein an increase in the concentration of the tested juice (from 2 to 40 µL/mL) enhanced the average absorbance in the MTT test (Table 1). This effect was detected in the treatments with the integral and natural juices of conventional and organic grapes, except for the integral juice produced with organic grapes treated with UV-C. Even for the integral juice of conventional and organic UV-untreated grapes, the natural juice of UV-untreated organic grapes, and the natural juice of conventional grapes treated with UV-C, the highest concentrations tested showed an average absorbance that was significantly higher than that of the smallest concentrations within the same group (Table 1).

**Figure 3:**
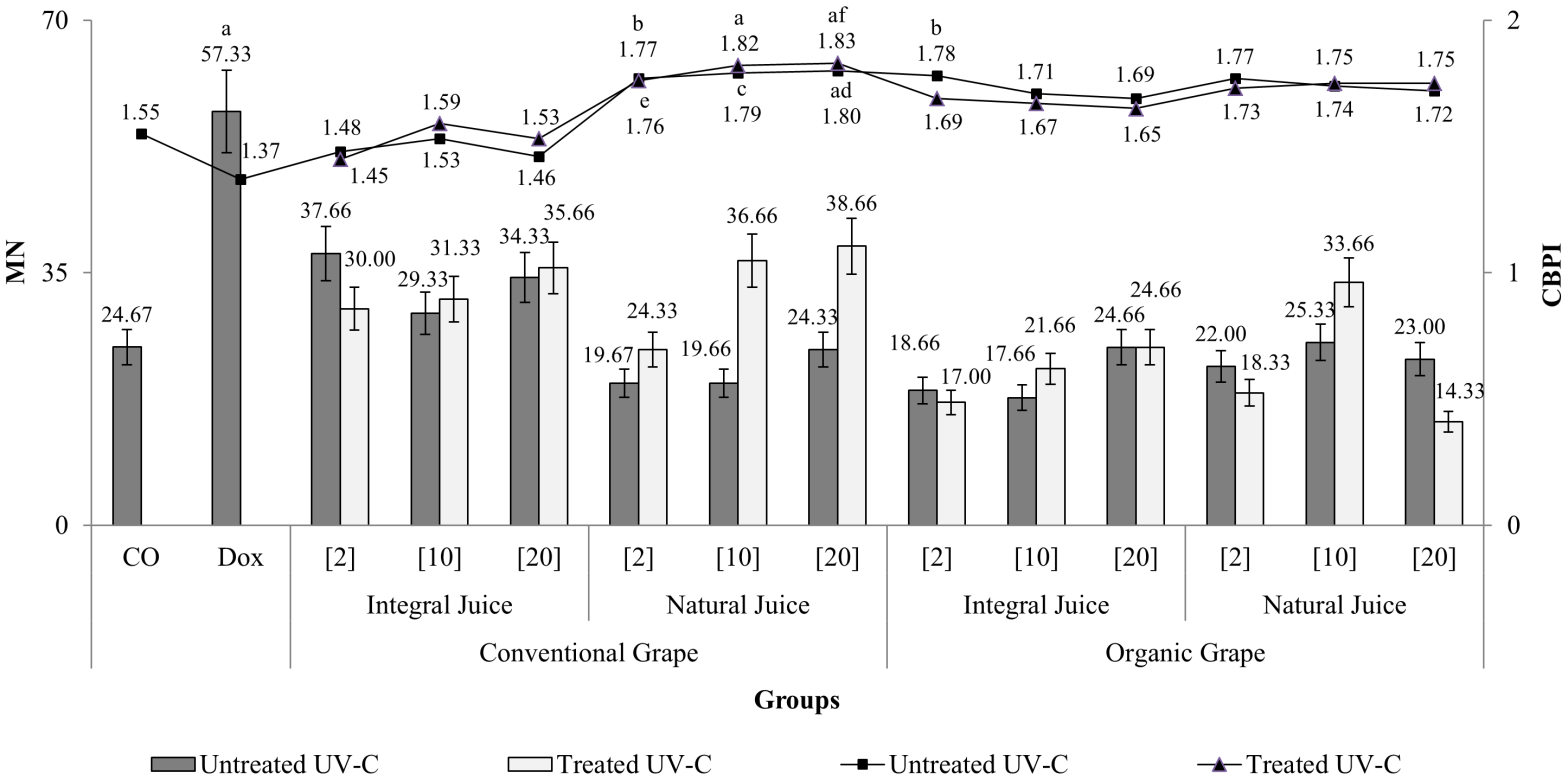
Average and standard deviation of the number of micronucleated cells (MN) (columns) and of the cytokinesis blocked proliferation index (CBPI) (line) for HTC cells treated for 24 hours. Negative control group (CO): 20 µL PBS/mL; Doxorubicin (Dox): 0.2 µg/mL; Integral and natural juice of conventional and organic grapes treated or untreated with UV-C: 2, 10 and 20 µL/mL. n = 3; 10^6^ cells per flask. a Statistically significant result relative to the negative control. b Statistically significant result relative to the treatment with 2 µL/mL integral juice of UV-untreated conventional grapes. c Statistically significant result relative to the treatment with 10 µL/mL integral juice of UV-untreated conventional grapes. d Statistically significant result relative to the treatment with 20 µL/mL integral juice of UV-untreated conventional grapes. e Statistically significant result relative to the treatment with 2 µL/mL integral juice of UV-C-treated conventional grapes. f Statistically significant result relative to the treatment with 20 µL/mL integral juice of UV-C-treated conventional grapes.

The stimulation of proliferation in HTC cells treated with grape juice was confirmed by the average CBPI values (Figure 3). The CBPI values obtained with natural juices of conventional grapes and with integral and natural juices of organic grapes were higher than those obtained with the negative control. Consequently, cytostasis was negative for these groups, indicating stimulation of cell proliferation by 18.18% to 50.90%. In fact, treatment with 20 µL/mL natural juice from conventional UV-untreated grapes and with 10 and 20 µL/mL of this juice from UV-C-treated grapes showed significantly different results compared with the negative control. It is noteworthy that integral juices of conventional UV-C-treated and -untreated grapes had the lowest CBPI and presented the lowest values of soluble acids (sugars) in their physical and chemical composition (data published by Pinto [20]).

Regarding the differences between natural juice and integral juice (namely the juices produced by liquefying the grapes or using the technological process of extraction by steam distillation, respectively), the CBPI results (Figure 3) indicate that the natural juices induced higher cell proliferation than the integral juices of conventional grapes treated or untreated with UV-C. The physico-chemical data of Pinto [20] support these findings because the natural juices of conventional grapes had higher values than the integral juices of conventional grapes with respect to the levels of soluble acids, total phenolic compounds, anthocyanins and trans-resveratrol.

Only the antioxidant activity (EC50: the amount of sample required for 50% reduction of the initial concentration of 2,2-diphenyl-1-picrylhydrazyl, DPPH) of all the integral juices was higher than that of the natural juices (data published by Pinto [20]). These data are in agreement with the study of Vedana et al. [29], who concluded that the heat used in the juice production process on one hand promotes the destruction of anthocyanins and some phenolic compounds but on the other hand enhances the bioavailability of compounds with antioxidant activity due to the elevated temperature and discontinuity of the tissue [12], [30].

According to Knize et al. [31], when food products are subjected to high temperatures, such as those used in the extraction of integral grape juice by steam distillation, toxic substances may be produced. This effect was not observed in our study because none of the integral juices showed cytotoxic or mutagenic potential.

All juices evaluated in this study possess a high content of phenolic compounds and anthocyanins (natural juices) or high antioxidant activity (integral juices), according to Pinto [20]. Additionally, HTC cells treated with these juices show a low frequency of MN (Figure 3) because treatment with these juices at all concentrations yielded MN levels similar to that of the negative control.

The phenolic content and antioxidant activity of the tested juices may have provided protection against free radicals and lipid peroxidation and consequently from DNA damage [7], [8], resulting in non-mutagenic effects. Dani [32] also observed that low concentrations of juices from *Vitis labrusca* cultivars Niagara and Bordô were non-mutagenic using a gene mutation assay in *Saccharomyces cerevisiae*. Lluís et al. [33] showed that extracts of grape seed and skin have no mutagenic potential by the micronucleus test using erythrocyte cells of Wistar rats treated for 72 hours; by the Ames test with the TA1535, TA1537, TA98 and TA100 strains at concentrations below 5 mg/plate; and by the chromosome aberration test in cultured human peripheral lymphocytes. Furthermore, in agreement with our results, data from Dani [34] also showed no significant differences in the mutagenic effects of juices produced from grapes from organic and conventional farming systems.

However, in the present study, the average MN level was lower (although not in a statistically significant way) in the juices produced from organic grapes than in those produced from conventional grapes. Treatment with juice from conventional grapes induced a non-significant increase (from 21.60% to 56.70%) in the production of micronuclei by the HTC cells compared with the negative control. Consistent with these results, the CBPI values (Figure 3), which indicate the cellular proliferation levels, were significantly higher upon treatment with 2 µL/mL integral juice from organic UV-untreated grapes than with the same concentration of integral juice from conventional UV-untreated grapes (conventional = 1.48±0.02; organic = 1.78±0.05). These effects may be due to the presence of some agrochemical residues in conventional juices and/or to the higher levels of soluble acids and phenolic compounds in organic integral juice relative to conventional integral juice, which may have stimulated proliferation and reduced the frequency of micronucleated cells.

The results of Dani et al. [34] support our findings because they claim that the biological activity of juices processed from *Vitis labrusca* grapes is influenced not only by the content of phenolic compounds but also by the agricultural management system used. In general, this study aligns with those that highlight the benefits of consuming organic food, which has more bioactive compounds [14], is free of agrochemical residues [35] and is more efficiently metabolized in cells.

According to Charles et al. [25], the application of conventional organic techniques and technology may alter the intrinsic levels of toxic substances in food. Accordingly, the cytokinesis blocked micronucleus assay (Figure 3) showed that with conventional grapes, almost all the integral juices had higher average MN levels than the natural juices. Furthermore, these integral juices caused less stimulation of cell proliferation (Figure 3) and lower levels of soluble acids, phenolic compounds, anthocyanins and trans-resveratrol (data published by Pinto [20]) compared with natural juices.

Thus, the thermal processing used in the production of integral juice may have resulted in the formation of mutagenic substances, the modification of protein function, the modification of tissues or the formation of free radicals, as indicated by Shibao and Bastos [36]. Ekasari et al. [37] also showed that fresh orange juice had no mutagenic properties but that mutagenicity was induced by the heat treatment during juice extraction.

The post-harvest treatment of grapes with UV-C did not alter cytotoxicity (Table 1), proliferation or mutagenicity (Figure 3) in the HTC cells. The UV-C irradiation of post-harvest grapes has a beneficial effect due to enhancement of the synthesis of total phenolic compounds and anthocyanins as well as the antioxidant capacity (EC50), as shown by Pinto [20]. It is thus significant that in the present study, this irradiation did not lead to the formation of cytotoxic or mutagenic compounds.

## Conclusions

This study reinforces the notion that the consumption of grape (*Vitis labrusca* L.) juice has no cytotoxic or mutagenic potential and suggests that the this juice, both in its natural form and from grapes of the organic production system, provides greater benefits to the cells and the DNA and hence to the body. This study supports the application of food production techniques that add value and nutrition to grape juice and promote its production, marketing and consumption by individuals.
